# Shrinking body sizes in response to warming: explanations for the temperature–size rule with special emphasis on the role of oxygen

**DOI:** 10.1111/brv.12653

**Published:** 2020-09-22

**Authors:** Wilco C.E.P. Verberk, David Atkinson, K. Natan Hoefnagel, Andrew G. Hirst, Curtis R. Horne, Henk Siepel

**Affiliations:** ^1^ Department of Animal Ecology and Physiology, Institute for Water and Wetland Research Radboud University Heyendaalseweg 135 6525 AJ Nijmegen The Netherlands; ^2^ Department of Evolution, Ecology and Behaviour University of Liverpool Liverpool L69 7ZB U.K.; ^3^ Faculty of Science and Engineering, Ocean Ecosystems — Energy and Sustainability Research Institute Groningen University of Groningen Nijenborgh 7 9747 AG Groningen The Netherlands; ^4^ School of Environmental Sciences University of Liverpool Liverpool L69 3GP U.K.; ^5^ Centre for Ocean Life, DTU Aqua Technical University of Denmark Lyngby Denmark

**Keywords:** Bergmann's rule, cell size, climate warming, gigantism, growth trajectory, hypoxia, life‐history trade‐off, phenotypic plasticity, temperature–size rule, thermal reaction norms

## Abstract

Body size is central to ecology at levels ranging from organismal fecundity to the functioning of communities and ecosystems. Understanding temperature‐induced variations in body size is therefore of fundamental and applied interest, yet thermal responses of body size remain poorly understood. Temperature–size (T–S) responses tend to be negative (e.g. smaller body size at maturity when reared under warmer conditions), which has been termed the temperature–size rule (TSR). Explanations emphasize either physiological mechanisms (e.g. limitation of oxygen or other resources and temperature‐dependent resource allocation) or the adaptive value of either a large body size (e.g. to increase fecundity) or a short development time (e.g. in response to increased mortality in warm conditions). Oxygen limitation could act as a proximate factor, but we suggest it more likely constitutes a selective pressure to reduce body size in the warm: risks of oxygen limitation will be reduced as a consequence of evolution eliminating genotypes more prone to oxygen limitation. Thus, T–S responses can be explained by the ‘Ghost of Oxygen‐limitation Past’, whereby the resulting (evolved) T–S responses safeguard sufficient oxygen provisioning under warmer conditions, reflecting the balance between oxygen supply and demands experienced by ancestors. T–S responses vary considerably across species, but some of this variation is predictable. Body‐size reductions with warming are stronger in aquatic taxa than in terrestrial taxa. We discuss whether larger aquatic taxa may especially face greater risks of oxygen limitation as they grow, which may be manifested at the cellular level, the level of the gills and the whole‐organism level. In contrast to aquatic species, terrestrial ectotherms may be less prone to oxygen limitation and prioritize early maturity over large size, likely because overwintering is more challenging, with concomitant stronger end‐of season time constraints. Mechanisms related to time constraints and oxygen limitation are not mutually exclusive explanations for the TSR. Rather, these and other mechanisms may operate in tandem. But their relative importance may vary depending on the ecology and physiology of the species in question, explaining not only the general tendency of negative T–S responses but also variation in T–S responses among animals differing in mode of respiration (e.g. water breathers *versus* air breathers), genome size, voltinism and thermally associated behaviour (e.g. heliotherms).

## I. INTRODUCTION: THE IMPORTANCE OF TEMPERATURE–SIZE RELATIONSHIPS

Body size is central to ecology at multiple scales, from organismal fecundity to the functioning of communities and ecosystems (Hildrew, Raffaelli, & Edmonds‐Brown, 2007). Larger individuals can potentially produce more offspring, live longer, may be superior competitors and be better at avoiding predators. These advantages favour growing to a large size (Brown & Sibly, 2006). The drawbacks to becoming large are varied. For example, growing larger takes more time and, during this time period, organisms may die or the environment may become unfavourable (Blanckenhorn, 2000). Larger individuals also commonly require more resources per unit time. Consequently, there is an optimal size and age to reproduce, which depends on the environmental conditions that enable growth and, for example, influence juvenile and adult mortality risks (Stearns, 1992).

**Fig 1:**
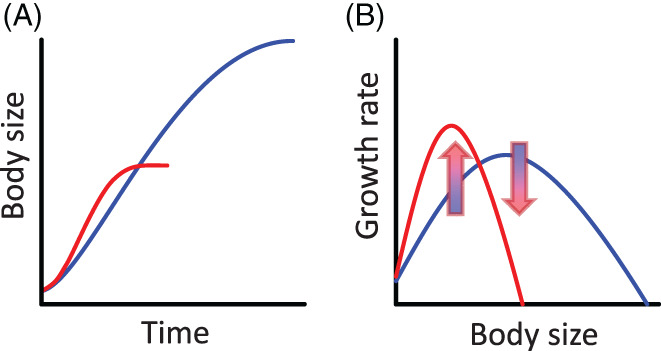
Thermal responses in body size (A) and growth rate (B). Responses are indicated for warm (red lines) and cold (blue lines) conditions. Arrows in B indicate that effects of warming are contingent on body size (and hence on time during ontogeny), stimulating growth during small, early life stages (upward arrow), but reducing growth in later, larger life stages (downward arrow). Note that this is a simplified schematic and in reality, the temperature–size rule (TSR) may progress irregularly over ontogeny (see Forster, Hirst, & Atkinson, 2011; Horne *et al*., 2019).

Over 80% of ectothermic species examined follow the temperature–size rule (TSR), that is they mature at a smaller size when reared in warmer conditions, despite initially growing faster (Atkinson, 1994; Fig. 1A). Despite the generality of this empirical pattern (Berrigan & Charnov, 1994), explaining it from life‐history theory is not straightforward (Atkinson & Sibly, 1997*b*; Day & Rowe, 2002). In fact, life‐history optimality models commonly predict that faster growth would favour animals growing to a larger size, and this is also generally observed when growth rates are experimentally manipulated by altering food quantity or quality (Kindlmann, Dixon, & Dostalkova, 2001; Diamond & Kingsolver, 2010; Yasuda *et al*., 2016). However, warming‐induced reductions in body size are pervasive (Daufresne, Lengfellner, & Sommer, 2009) and have been termed the third universal response to warming (Gardner *et al*., 2011); the first and second universal responses to warming being directed dispersal in space (range shifts) and in time (phenological shifts). Clines in body size are observed across thermal geographic gradients (e.g. latitude or altitude), where small body size is typically associated with warmer conditions (low latitude or altitude) and such clines are referred to as Bergmann's rule for differences among closely related species, and as James’ rule for differences among populations of the same species. The TSR is restricted to phenotypically plastic effects that arise during ontogeny, setting it apart from James’ and Bergmann's rules, which can include ecological and evolutionary body size responses to temperature and associated climatic factors over longer timescales [see Watt, Mitchell, & Salewski, 2010 and Pincheira‐Donoso, 2010 for in‐depth discussions on James’ rule and Bergmann's rule and their applicability to ectotherms].

There is great interest in solving the life‐history puzzle of the TSR, not least because more than 99.9% of all species are ectotherms. Previous research on the TSR has focussed on whether there is a general mechanism to explain the TSR and whether the TSR is adaptive. Although the idea of a general explanation makes intuitive sense when confronted with a pattern that is so pervasive, a simple, general explanation has not yet emerged. The finding that size reductions with warming can be achieved at different levels of organization and stages of ontogeny, and by different mechanisms (e.g. thermal responses in cell size, offspring size, differences in thermal sensitivity of growth rate and development rate), has in itself been used to argue that the TSR is adaptive (Atkinson, 1994; Forster & Hirst, 2012). In addition, similar directions of plastic and evolved thermal responses (e.g. both becoming smaller in the warm), and of latitudinal *versus* plastic responses, suggest that the TSR is likely to be adaptive (Partridge *et al*., 1994; Kingsolver & Huey, 2008; Horne, Hirst, & Atkinson, 2015). To understand the complex nature of thermal adaptation and the TSR better, Angilletta & Dunham (2003) advocated a multivariate approach with greater emphasis on the ecological context in which life histories evolve within physiological constraints set by their body plan. Similarly to a recent review we highlight the role of oxygen (Audzijonyte *et al*., 2019), but we here emphasize not just temperature–size (T–S) responses induced by oxygen limitation but also how T–S responses can have evolved to avoid such limitation. Additionally, we adopt a broader focus beyond aquatic ectotherms to include terrestrial ectotherms. We first describe what constitutes the TSR. Next, we summarize the observed variation in the strength of the T–S response across groups of organisms. We then proceed to discuss how T–S responses can arise from thermal influences on growth and development rates, and the adaptive value of maturing at a certain size and age (Table 1). Past reviews have focussed on whether a species follows the rule or not (Shelomi, 2012; see also Blanckenhorn & Demont, 2004), but we consider that quantifying differences in the strength of the T–S response across groups of organisms will more likely reveal the relative contributions of different explanations for T–S responses. Understanding the causes of variation in the magnitude of T–S responses may lead to a more complete explanation of why a reduction in body size with warming (the TSR) is especially prevalent. We conclude this review by suggesting research that would best advance our knowledge of temperature effects on body size.

**Table 1:** Overview of the different mechanisms, grouped into mechanistic and evolutionary explanations. See text for further details

Explanations	Further reading
**Mechanistic (proximate) explanations**	
Animals grow faster but develop even faster in warm conditions	
– Different thermal sensitivity of DNA replication *versus* protein synthesis: DNA replication (limited by enzyme kinetics) is more sensitive to temperature than protein synthesis (limited by diffusion)	Section IV.1
– At high temperatures or low oxygen, animals may preferentially allocate resources towards development and away from growth	Section IV.5
– Thermal sensitivity of growth may be reduced to prevent oxygen limitation, whereas thermal sensitivity of development may depend on genome size	Sections IV.1, IV.3 and IV.7; Fig. 4
Larger requirements for resources (food, oxygen) in warmer conditions prevent animals from growing larger	
– Different thermal sensitivity of catabolism and anabolism: growth efficiency is lower in warmer conditions	Section IV.3
– Different thermal sensitivity of size‐dependent changes in catabolism and anabolism: decline in growth efficiency with size is amplified in warmer conditions, resulting in a lower growth efficiency in warmer conditions for large (but not small) individuals	Section IV.3
– Insufficient capacity to extract oxygen constrains animals from growing larger, even more so under warm conditions	Section IV.2
Animals consist of smaller cells in warm conditions	
– A large genome (resulting in a larger cell size) with multiple copies ensures sufficient enzyme activity in cold conditions	Section IV.7
– Smaller cells have more membrane surface area relative to their volume supporting a greater capacity for oxygen transport in warm conditions	Section IV.7
– The ratio between oxygen supply and demand may function as a threshold for cell growth, thus regulating cell size and possibly the critical size observed in insects	Section IV.7
**Evolutionary (ultimate) explanations**	
It becomes more advantageous to grow larger in cold conditions because of reduced mortality	
– Senescence and mortality are greater in warmer environments, favouring early maturation (at a smaller size)	Sections V.1 and V.3
It becomes more advantageous to grow larger in cold conditions because of gains in fecundity	
– Fecundity may increases more strongly with body size in cold conditions, favouring large size	Sections V.1 and V.3
It becomes more advantageous to grow larger in cold conditions because of resource limitations	
– Selection for starvation resistance typical for larger animals is stronger in cool conditions	Sections V.2 and V.3
It becomes more advantageous to produce an additional generation rather than growing to a larger size in growing populations	
– Faster maturity (at a smaller size) allows for completion of an additional generation in multivoltine species	Section VI.1; Fig. 5.
The ‘Ghost of Oxygen‐limitation Past’ has led to the evolution of thermal reaction norms for adult size that are anticipatory to temperature and oxygen conditions experienced by ancestors	
– Past occurrences of oxygen limitation have selected for a canalized response with smaller sizes under warmer conditions as a compensatory response to safeguard sufficient oxygen provisioning	Sections IV.2, VI.3 and VI.4

## II. THE NATURE OF THE TSR

The TSR in its simplest form describes how ectotherms develop to a smaller size for a given stage, especially late in ontogeny (e.g. size at maturity), when reared under warmer conditions. Size‐at‐stage results from the interplay between the rate of growth and the length of the period spent growing, and therefore a faster growth to a smaller size in the warm (i.e. the TSR) arises logically from warming stimulating development rate more than growth rate. Body size responses to temperature vary both in strength and sign (i.e. increases or decreases) across species. Consequently, the field has moved to a more quantitative approach examining the magnitude and direction of size responses to temperature (e.g. Forster, Hirst, & Atkinson, 2012) rather than adopting a binary classification of whether a species is smaller or larger at a given ontogenetic stage when reared in warmer conditions. In addition, most of the literature focuses on size at maturity, but for organisms with indeterminate growth, T–S responses can differ between size at maturity and asymptotic size, suggesting that different mechanisms are involved (Hoefnagel *et al*., 2018). T–S responses of eggs are also somewhat different (weaker) than those for size at maturity (Atkinson *et al*., 2001). The T–S response can change as animals proceed through ontogeny, but in a discontinuous fashion, being more pronounced in certain larval instars than others (Forster, Hirst, & Atkinson, 2011; Forster & Hirst, 2012; Horne *et al*., 2019). TSR patterns may arise not only during ontogeny, but also across sequential generations, which develop at different temperatures in seasonal environments (e.g. summer and winter generations in the field) (Horne, Hirst, & Atkinson, 2017). Moreover, such T–S responses may also be observed across populations of a species, with latitudinal clines in adult body size also broadly matching plastic body‐size responses to rearing temperatures (Horne *et al*., 2015). Size reductions in response to warming are also evident across species within whole communities (Daufresne *et al*., 2009). Although the mechanisms generating T–S patterns within and across species could be different, the overall trends do indicate a size‐based filtering that favours smaller species and/or younger ages, as has been observed along a latitudinal thermal cline (Zeuss, Brunzel, & Brandl, 2017) and along a thermal gradient associated with urbanization (Merckx *et al*., 2018). This review focuses on plastic body‐size responses to temperature. However, given the concordance between the TSR, James’ rule and Bergmann's rule, we also discuss explanations with an ecological and evolutionary basis, where temperature is involved only indirectly (e.g. as a cue for seasonal progression and for time remaining to complete development).

Finally, the TSR is only a puzzle when evaluated under benign conditions, including non‐stressful temperatures and non‐limiting resource supply (Atkinson, 1994; Walczyńska, Kiełbasa, & Sobczyk, 2016). For example, when high temperatures impair growth, rather than stimulate it, life‐history theory predicts animals to mature at a smaller size. Similarly, when warming alleviates cold, stressful temperatures, it may result in animals growing to a larger body size (Forster, Hirst, & Woodward, 2011). Ectotherms that follow the TSR, grow faster but to a smaller size in warmer conditions. Therefore, effects of temperature on growth differ throughout ontogeny: at earlier or smaller life stages temperature stimulates growth while at later or larger life stages temperature reduces growth (Fig. 1B). Thus, understanding the effects of temperature on size needs to incorporate interactions between time, temperature and body size.

## III. PATTERNS IN T–S RESPONSES

### (1) T–S responses due to phenotypic plasticity

**Fig 2:**
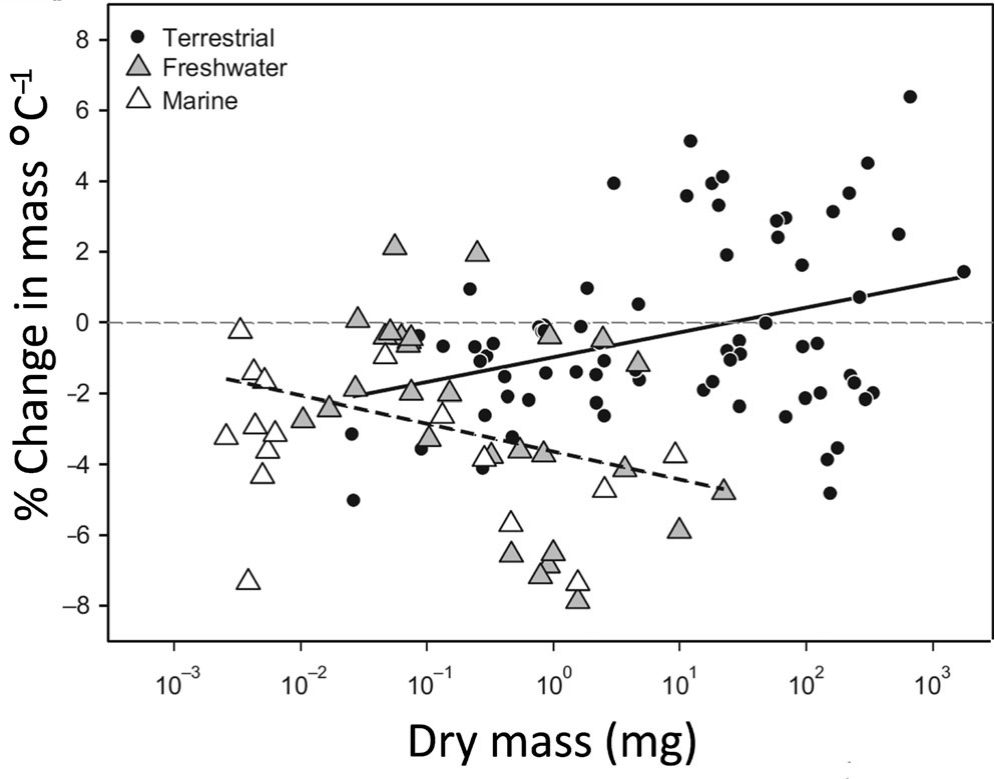
Temperature–size (T–S) responses (% change in body mass per °C) for terrestrial (black circles), freshwater (grey triangles) and marine (white triangles) arthropod species, plotted against their dry mass (standardized to 20°C) With increasing body mass, T–S responses became more negative in aquatic arthropods (dashed line; *F*_1,43_ = 5.40, *P* = 0.02, *r* ^2^ = 0.09), but in terrestrial arthropods they became more positive (solid line; *F*_1,69_ = 9.28, *P* = 0.003, *r* ^2^ = 0.11). Figure reprinted from Horne *et al*. (2015) with permission from John Wiley & Sons Ltd/CNRS.

Although adult body size is usually reduced under warmer rearing conditions (i.e. following the TSR), we will also describe the substantial variation in responses across different taxa and environments (terrestrial, aquatic). Taking a meta‐analytical perspective, Horne *et al*. (2015) extended the work of Forster *et al*. (2012) and Klok & Harrison (2013), to find distinct patterns in the extent to which body size responds to temperature across taxonomic groups of arthropod species. A primary finding was that T–S responses became more negative (stronger TSR) in aquatic arthropods with increasing body size (Fig. 2). In terrestrial arthropods, this pattern with body size appeared to be reversed. However, body size and voltinism (i.e. the number of generations of an organism in a year) tend to co‐vary (univoltine species, with one generation per year, are typically larger than multivoltine species, which have more than one generation per year). In terrestrial arthropods, voltinism has been found to be a stronger predictor than body size, with univoltine species often displaying the reverse T–S response, such that they commonly mature at a larger size in the warm. Thus, within aquatic arthropods, the T–S response appears to become stronger with increasing body size, whereas within terrestrial arthropods the opposite pattern is found, with the T–S response weakening and eventually reversing with increasing body size (Fig. 2).

### (2) T–S responses across populations, species and communities

Horne *et al*. (2015) report a concordance between phenotypically plastic size responses to temperature (the TSR) and latitudinal clines in body size (i.e. James’ rule). Obviously, latitudinal size clines could be related to various factors other than temperature, which also co‐vary with latitude (e.g. duration of growth season, day length, food availability, potential evapotranspiration, and thermal fluctuations), and the mechanisms could likewise differ as they apply to differences across populations. For example, dispersal could obscure spatial relationships between environmental temperature and body size (Horne, Hirst, & Atkinson, 2018), as has been suggested for altitudinal clines in body size within several species of grasshoppers differing in dispersal potential (Levy & Nufio, 2015) and latitudinal clines across dytiscid beetle species (Pallarés *et al*., 2019). Still, the correspondence noted by Horne *et al*. (2015) suggests that these body‐size responses across individuals, populations and species may share at least some of the same temperature‐related drivers. This makes it informative to compare T–S responses at the population and species level across aquatic and terrestrial groups of different body size.

Makarieva, Gorshkov, & Li (2005) showed that the largest terrestrial ectotherm species tend to live in the warm tropics. By contrast, in a variety of animal groups, aquatic species of gigantic proportions have been documented in cold, polar regions (Moran & Woods, 2012). These contrasting geographical trends in maximum body size can be seen as a special case of the more general pattern in which T–S responses across latitudinal clines become increasingly negative in larger‐bodied taxa in aquatic but not terrestrial habitats. Similarly, among aquatic amphipod communities, stronger T–S responses were observed for the largest species (a sixfold change), while changes in median body size were less pronounced (2.6‐fold change) (Chapelle & Peck, 2004). In summary, the pattern of intraspecific T–S responses becoming stronger with increasing body size in water but not on land is also observed across species and across communities. This concordance across ecological levels of organization could be a coincidence or could reflect similar drivers and constraints.

## IV. THE DEPENDENCY OF T–S RESPONSES ON GROWTH AND DEVELOPMENT

### (1) Growth and development rates have different thermal sensitivities

Differences in thermal sensitivity of growth and development rates give rise to T–S responses (Forster, Hirst, & Woodward, 2011; Banas & Campbell, 2016; Hoefnagel *et al*., 2018), and many explanations therefore focus on explaining differences in the thermal sensitivity of growth and development (Table 1). Instead of differences in their thermal dependency, Walters & Hassall (2006) argued for a focus on differences between the minimum threshold temperature for growth and that for development (i.e. the temperature below which growth and development are arrested). Indeed, when growth and development rates change linearly with temperature, a decrease in the ratio between growth rate and development rate with warming is equivalent to a greater threshold temperature for development rate than for growth rate. However, different threshold temperatures for growth and development are not a necessary condition for T–S responses to arise when thermal dependencies are non‐linear (e.g. Forster, Hirst, & Woodward, 2011; see also Kutcherov, Lopatina, & Kipyatkov, 2011; Sweeney *et al*., 2018).

**Fig 3:**
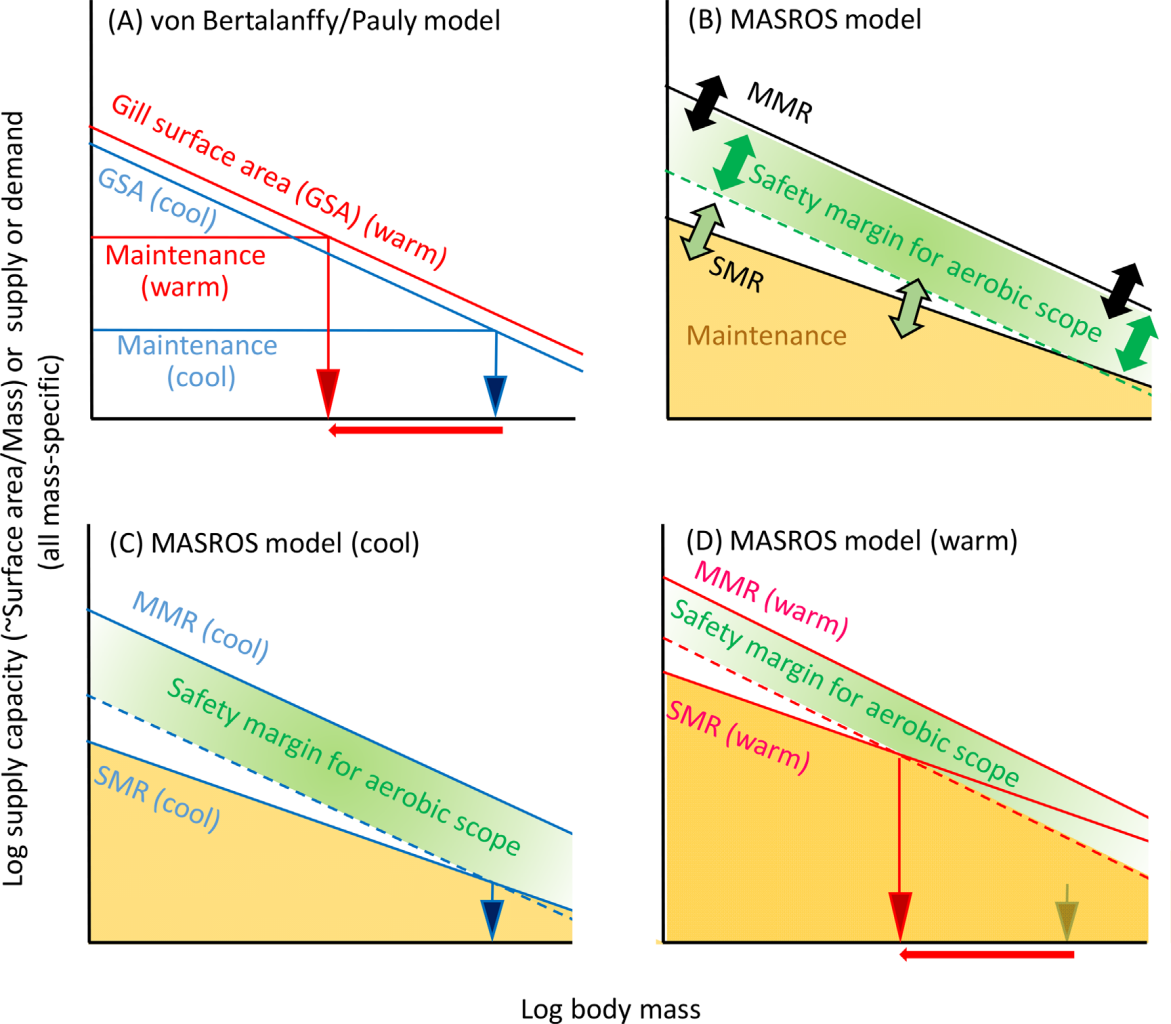
Role of constraints in the von Bertalanffy/Pauly model (A) and the maintain aerobic scope and regulate oxygen supply (MASROS) model (B–D). In A, constraints on growing to a larger size are considered to be insurmountable, arising from geometric constraints on gill surface area scaling, and growth ceases when maintenance metabolism converges to supply capacity. Maintenance is here considered to fuel essential processes such as maintenance of electrochemical gradients, protein synthesis, and repair. In the MASROS model, animals still have aerobic scope left when reaching maximum size, which is considered to be a safety margin when animals face demanding but transient conditions (e.g. disease, episodes of hypoxia, predator attack, and possibly part of reproduction). Aerobic scope not reserved for the safety margin (in white) can be used to fuel growth and other routine activities (e.g. activity, digestion and possibly part of reproduction). Evolution is thus assumed to have modified growth trajectories to avoid oxygen limitation. Growth trajectories can be modulated by adaptive changes in the scaling of standard metabolic rate (SMR), maximum metabolic rate (MMR) or the width of the safety margin for aerobic scope. Warm conditions (shown in red), may lead to growth to a smaller size if the thermal sensitivity of maintenance (SMR) is higher than that of supply (MMR). This size decrease could be partly compensated for by allowing a reduction in the safety margin (panel D). Note that the slopes of the lines (i.e. the scaling exponents) can also vary with temperature, but are here kept constant for reasons of clarity.

Van der Have & de Jong (1996) suggested that protein synthesis, involved in both cell and organism growth, is limited by relatively temperature‐insensitive diffusion of heavy ribosomal sub‐units, and that DNA replication – central to cell division, differentiation, and rate of organismal development towards maturity – is instead limited by the more thermally sensitive rates of DNA polymerase activity. Consequently, they argued that DNA replication (related to differentiation) is more temperature sensitive than protein synthesis (related to growth), thereby linking whole‐organism growth and development to the kinetics of individual enzymes. This mechanism may contribute to producing negative T–S responses. However, given the extant variation in the strength of the T–S response across different groups of taxa and during ontogeny (see Section III) differences in thermal sensitivity can be modulated (see also Section IV.2). Modulation of the thermal sensitivity of growth and development is perhaps most obvious in unicells (with binary division). Since the offspring size is half that of the parent cell, the ratio between specific growth rates and development rates equals 2 or they would increase or decrease in body size *ad infinitum*. Hence, they can only achieve a T–S response if temperature shifts the ratio between growth rates and development rates away from 2 temporarily (Forster, Hirst, & Atkinson, 2011; Forster, Hirst, & Esteban, 2013). Thus, a greater thermal sensitivity of development rate relative to growth rate may arise partly because of differences in kinetics of DNA replication and protein synthesis, but other additional explanations are required to explain the variation in T–S responses.

### (2) Are there insurmountable constraints on growth?

Debates continue on whether or not growth rate is increasingly constrained during ontogeny, and if so, whether or not warmer temperature increases these constraints, leading to smaller size at maturity or final size (Pauly, 1998; Lefevre, McKenzie, & Nilsson, 2017; Pauly & Cheung, 2018; Audzijonyte *et al*., 2019). The debated constraint is geometric, based on reductions in the surface area to volume ratio as size increases, which has been called a ‘dimensional tension’ by Pauly & Cheung (2018). The diminishing ratio of surface area to volume has been argued to result in resource limitation – food limitation owing to insufficient area of the digestive tract, or oxygen limitation owing to insufficient area of respiratory surfaces (Kooijman, 2010; Pauly, 2010). Oxygen limitation has been emphasized in aquatic species, which can expend non‐trivial proportions of their energy budget obtaining oxygen (Von Bertalanffy, 1960; Pauly, 2019). This geometric constraint is used to explain growth deceleration during ontogeny up until maximum size where growth is no longer possible. At this point, the supply of resources available for growth and other routine metabolism [upper line at each temperature in Fig. 3A, corresponding to ‘anabolism’ of Von Bertalanffy, 1960 and assimilation of Kooijman, 2010] has converged with the line representing non‐growth or ‘maintenance’ resource demand [lower line at each temperature in Fig. 3A, corresponding to ‘catabolism’ of Von Bertalanffy, 1960]. Added to the dimensional tension is the idea that resource uptake has a rate‐limiting step that is less sensitive to temperature (e.g. diffusion) than is resource demand (e.g. rate of enzymatic reactions). Consequently, maximum resource supply increases relatively little as temperatures rises (from blue to red), compared with the greater increase of maintenance costs. Oxygen diffusion in water is relatively temperature insensitive, accelerating only by about 10% with 10°C warming (Verberk *et al*., 2011), contrasting with the approximate doubling of metabolic rate (Seebacher, White, & Franklin, 2015). However, the uptake of food resources is less likely to be widely thermally insensitive, varying with feeding mode (Dell, Pawar, & Savage, 2014) and how temperature affects food availability (i.e. the balance of food production to consumption).

Constraints on oxygen supply have been described as ‘insurmountable’ (Pauly, 1998; Lefevre *et al*., 2017) or ‘uncircumventable’ (Pauly & Cheung, 2018). Yet they are not completely insurmountable. Organisms have evolved the capacity to: (*i*) increase surface area for resource uptake during growth, such as by changing body shape (Hirst, Glazier, & Atkinson, 2014; Glazier, Hirst, & Atkinson, 2015) or increasing the size of uptake organs (Antoł *et al*., 2020) or their surfaces (gill re‐modelling; Nilsson, Dymowska, & Stecyk, 2012); (*ii*) increase rates at which they obtain and distribute resources (e.g. by increasing feeding activity or by active ventilation and circulation; Woods & Moran, 2020); and (*iii*) reduce rates of demand for resources (e.g. less locomotion, or lower mitochondrial density). These adjustments to the rate of resource uptake and demand reduce the likelihood that the constraints will be observed directly in controlled laboratory studies of the TSR, which provide abundant food, levels of oxygen availability typical for the species, non‐extreme (‘physiological range’) temperatures and an absence of predators and disease. Even in the field, such constraints may be observed only occasionally. On the other hand, overcoming or avoiding these physical constraints is unlikely to be cost‐free. Thus, organisms may have adapted so that they are not ‘panting for breath’ during normal growth, but could nonetheless experience resource limitation under more demanding conditions (e.g. when pursued by predators or encountering pathogens). Although such demanding conditions may be rare, they are also disproportionately detrimental. Organisms should therefore maintain a safety margin (e.g. aerobic scope) to prevent resource limitation of growth and reduction in fitness, exemplified by the maintain aerobic scope and regulate oxygen supply (MASROS) model, in which size is adjusted to maintain sufficient oxygen supply relative to demand (Atkinson, Morley, & Hughes, 2006; Fig. 3B). Selection to avoid resource limitation may favour adaptive modulation of growth in response to temperature especially when temperature is a reliable cue (i.e. temperature has correlated with fitness benefits from developing faster or maturing at a smaller size during the population's evolutionary history). This adaptive response to *avoid* resource limitation or other harm represents an important conceptual distinction. Instead of direct constraints on growth, we here emphasize the evolution of adaptive reaction norms in which growth responds to temperature as a cue to avoid harm.

Evolutionary adjustments (double‐headed arrows in Fig. 3B) can be made to resource uptake capacity (upper boundary of safety margin), to the size of the safety margin (height of green shaded area at different body sizes), and to the amount of other non‐growth investment, sometimes referred to as ‘maintenance’ (height of orange shaded area at different body sizes). All of these can shape the resulting growth trajectories. Thermal responses in growth trajectories are the evolutionary outcome that temperature has had on these factors (Fig. 3C and D). This adaptive perspective should also be applicable to other potential constraints affecting the evolution of the TSR (e.g. temperature‐dependent uptake of food resources, whose safety margin is set by extra feeding and assimilation capacity and by the amount of stored reserves; or by viscosity affecting oxygen supply; Verberk & Atkinson, 2013). In summary, simultaneous adaptive modulation of growth, maintenance and a safety margin reflects the evolutionary effects of past size‐ and temperature‐dependent constraints on resource availability and other selection pressures. Evolved plastic responses of growth to temperature can mitigate current or predictable future resource limitations, thereby avoiding constraints on whole‐organism growth. Adaptive modulation of growth trajectories likely also integrates other fitness‐enhancing activities such as reproductive development or reproduction, leading to a deceleration of growth with increasing body size (Kozłowski, Czarnołęski, & Danko, 2004; Kooijman, 2010; Marshall & White, 2019).

### (3) Thermal responses in growth rate

In order to grow, organisms need resources such as food and oxygen, which together shape the energy budget of an organism. Changes in the energy budgets and energy allocation with temperature have been used to explain the TSR (e.g. Pauly, 2010; see Section IV.2). Much of this work can be traced back to the work of Von Bertalanffy (1960) and Pütter (1920) who noted that somatic growth must be equal to the difference between anabolism and catabolism, although part of the energy surplus must also be allocated to reproductive growth (Kozłowski *et al*., 2004; Marshall & White, 2019). If catabolism increases relative to anabolism with increasing body mass, a decrease in body size with warming could then arise when temperature stimulates catabolism more than anabolism (Von Bertalanffy, 1960) or when temperature stimulates resource demand more than supply (DeLong, 2012; Fig. 3A). Angilletta & Dunham (2003) argued that while warming could increase absolute growth rates, warming must also, according to von Bertalanffy's growth model, reduce net growth efficiency (expressed as the percentage of biomass produced relative to total energy absorbed), as relatively more energy is spent on catabolism with warming. However, their analysis of published data on growth efficiency did not find the expected decrease in net growth efficiency with warming. A potential resolution to this problem is that the thermal dependency of net growth efficiency is itself size dependent. Consequently, the decline in growth rates and growth efficiency observed with increasing size should be more pronounced under high temperatures (e.g. Perrin, 1988; Panov & McQueen, 1998; Kozłowski *et al*., 2004; Hoefnagel *et al*., 2018). It has been suggested that larger organisms have smaller net energy balances in warm conditions because oxygen demand increases with temperature relative to oxygen supply (Pedersen, 1987; Pörtner, 2001; Pauly, 2010; Verberk *et al*., 2011; Verberk & Atkinson, 2013). However, whether the decline in growth rates and growth efficiency with increasing size is constrained by resource limitation is still debated (see Section IV.2). A role for oxygen in generating the TSR may explain the stronger T–S responses observed in aquatic taxa compared to terrestrial taxa (Forster *et al*., 2012; Horne *et al*., 2015; Rollinson & Rowe, 2018), owing to the greater challenges of breathing underwater (lower diffusion rates, larger costs of ventilation) (Dejours, 1981; Verberk *et al*., 2011; Verberk & Atkinson, 2013). Few studies have tested interactive effects of oxygen and temperature on growth and size at stage, but the few that have demonstrate that T–S responses depend on oxygen conditions in aquatic isopods (Hoefnagel & Verberk, 2015), and in air‐breathing fruit flies (Frazier, Woods, & Harrison, 2001). Size reductions with warming were more pronounced under hypoxia and less pronounced – or reversed – under normoxia and hyperoxia. This suggests either a direct role of oxygen in generating the TSR (i.e. the strongest T–S response is observed under conditions where resource limitation is most likely), or that oxygen limitation has acted as a selection pressure on growth trajectories, and animals use temperature and oxygen conditions as cues to modulate growth. Given that effects of hyperoxia are much weaker (but usually opposite) to those of hypoxia, oxygen limitation as a selection pressure on growth seems more likely. Indeed, direct evidence that individuals become more prone to warming‐induced oxygen limitation as they grow larger is scarce, and may differ between aquatic ectotherms (e.g. fish) and terrestrial ectotherms (e.g. insects), as the costs of increasing oxygen uptake are greater in water than in air (Verberk & Atkinson, 2013; Verberk & Bilton, 2013).

Fish appear to adhere to the TSR (see Section IV.4), but size dependency of oxygen supply capacity in fish has been debated (see Section IV.2). Since fish can dynamically alter their gill surface area, it is unlikely that constraints are completely insurmountable, but gill proliferation also carries costs, such as the cost of maintaining ion homeostasis and water transport, increased exposure to toxic substances in the water, and increased risk of disease and parasitism (Nilsson *et al*., 2012; Audzijonyte *et al*., 2019). Since excessive oxygen itself is toxic, the act of balancing toxicity and asphyxiation risks may also directly reduce performance of animals with an excess capacity for oxygen uptake (Verberk & Atkinson, 2013). To explain the TSR from an oxygen‐limitation perspective, these costs and benefits of altering capacity for oxygen uptake must be size and temperature dependent. Most studies focus on two‐way interactions, rather than the three‐way interaction between size, temperature and oxygen (Woods & Moran, 2020). Boundary layers at the gill surface affect uptake capacity in such a size‐ and temperature‐dependent manner; they result from viscosity and impede oxygen diffusion, especially in colder, more viscous water, and smaller animals are disproportionately affected (Verberk & Atkinson, 2013). Consequently, larger individual fish in warmer waters could have a lower aerobic scope or a higher sensitivity to oxygen limitation (J.G. Rubalcaba, W.C.E.P. Verberk, A.J. Hendriks, B. Saris & H.A. Woods, in preparation). There is also evidence that larger individuals are more prone to oxygen limitation in some fish species (Burleson, Wilhelm, & Smatresk, 2001; Robb & Abrahams, 2003; Reid *et al*., 2013), but it is difficult to generalize this to all fish, given the many different strategies for coping with hypoxia (Chapman & McKenzie, 2009). Indeed, fish may deal with hypoxic stress in a size‐dependent manner, with larger animals relying more on anaerobic metabolism (Goolish, 1989; Urbina & Glover, 2013; Lv *et al*., 2018). On the relatively short timescales typical for hypoxia‐tolerance assays, larger fish could supplement their energy needs with anaerobic metabolism; on longer timescales of growth and development, a lower aerobic scope of larger fish in warm waters could reduce growth. This is an area in need of more empirical data.

In terrestrial ectotherms such as many insect species, evidence that risks of oxygen limitation increase as they grow larger is scarce, possibly because animals can compensate in a range of ways (e.g. by increasing capacity for ventilation and circulation; see Harrison, Greenlee, & Verberk, 2018). In the grasshopper *Schistocerca americana*, hypoxia sensitivity (used here as a proxy for risks of oxygen limitation) was highest in the youngest instars which lack air sacs and rely more on diffusive gas exchange (Greenlee & Harrison, 2004). Larger individuals tend to employ convective gas exchange, which could explain their lower sensitivity to hypoxia. Also across species, there is little evidence for size dependency of hypoxia sensitivity. For example, Harrison, Klok, & Waters (2014) found the critical oxygen partial pressure (*p*O_2_) for metabolism to be independent of adult body size across a range of insect species. Larger species likely prevent progressive oxygen limitation with increasing body size by having greatly increased tracheal dimensions and these do appear to set upper limits to the size that insects may attain (Kaiser *et al*., 2007). In cases where oxygen limitation is less of a constraint on growth, patterns of larger species at higher temperatures have been explained by the need to maintain metabolism (expressed per gram of body tissue) within an optimal range, as increasing body size reduces metabolism, counteracting the increased metabolism associated with higher temperatures (Makarieva *et al*., 2005). In summary, in terrestrial ectotherms, several reasons may explain why sensitivity to low oxygen is decoupled from size, although upper size limits may still be set by limits to tracheal expansion.

If larger aquatic species are indeed more challenged to provision their tissues with adequate oxygen to maintain sufficient aerobic scope (see also Section IV.2), this would provide an explanation for stronger T–S responses with increasing body size in these taxa (Fig. 2; see also Section IV.4). Similarly, if larger terrestrial species are less challenged by oxygen limitation because of increased reliance on convective transport, this could also explain why T–S responses weaken and then reverse with increasing species body size in terrestrial arthropods (Fig. 2; Klok & Harrison, 2013).

### (4) Fish and the temperature–size rule

Fish have been documented to adhere to the TSR (e.g. Trexler, Travis, & Trexler, 1990; Dhillon & Fox, 2004; Loisel, Isla, & Daufresne, 2019), and thermal clines in the field are often related to size clines (Daufresne *et al*., 2009; Baudron *et al*., 2014; Van Rijn *et al*., 2017; Moffett *et al*., 2018; but see Belk & Houston, 2002). Latitudinal clines in fish body size have also been documented, but other factors may play a role here. For instance, larger fish may have a greater capacity to disperse to higher latitudes (Weber *et al*., 2015), increased mortality in warmer areas may select for individuals to mature faster at a smaller size (Heibo, Magnhagen, & Vøllestad, 2005), and warming may produce opposite effects in species with contrasting thermal niches (Rypel, 2014). Moreover, fishing pressure may greatly affect size distributions in the field, confounding, blurring or strengthening patterns in body size related to temperature (Cheung *et al*., 2013; Tu, Chen, & Hsieh, 2018).

Clearly, the existence of large species living in warm tropical waters indicates evolutionary capacity to overcome constraints on growth to a large size. Hence, these constraints are not insurmountable from an evolutionary perspective (see Section IV.2). Instead, adaptive evolution can enhance the capacity to supply oxygen depending on the lifestyle (Seibel & Deutsch, 2020). Different adaptations can enhance oxygen supply such as planktonic feeding with greatly enlarged gills (e.g. whale shark), ram ventilation (e.g. tuna, marlin) or adopting a sluggish lifestyle as adults (groupers). While large fish species such as those mentioned above can clearly live in warmer waters (an interspecific pattern), it is unknown whether they will show a stronger T–S response (an intraspecific pattern). They could grow larger still when reared under colder conditions, but such experiments would be logistically challenging: consequently, T–S responses for size at maturity are recorded only up to the size of small fish or large insects. A major issue is therefore predicting the extent to which strengthening T–S responses with species body size in aquatic ectotherms will extend further to include commercial fish and aquaculture species. A recent study indicated that responses in mean fish size to temperature were weakening and reversing towards larger sizes (Audzijonyte *et al*., 2020), although it is unclear how results on mean size relate to size at stage (e.g. maturity or maximum). Concordant with a role for oxygen, Van Rijn *et al*. (2017) focussing on maximum size in the field, found greater T–S responses in more active fish species. It is challenging, however, to isolate effects of temperature on body size in field data where responses could also reflect (size‐dependent) species interactions, dispersal, differences in productivity and length of the growing season. Therefore, rearing experiments under controlled conditions should help us understand physiological mechanisms better (Edeline *et al*., 2013; Ohlberger, 2013; Knouft, 2014).

### (5) Thermal responses in development rate

Effects of oxygen and food are not limited to growth rate, but may also act on development (Table 1). Callier & Nijhout (2011) showed that in growing caterpillars of *Manduca sexta*, the decision to moult or pupate is size and oxygen dependent. As animals increase their body mass, their demand for oxygen also increases, but since the tracheal system can only be enlarged upon moulting they cannot correspondingly increase the capacity for oxygen supply. Such low capacity for oxygen supply relative to demand triggers the endocrine cascade that advances development (see also Callier *et al*., 2013; Kivelä *et al*., 2018). Greenberg & Ar (1996) found that the mealworm beetle (*Tenebrio molitor*) developed into smaller adults when reared under hypoxia, but more than doubled the number of moults to get there compared to normoxia. Under hyperoxia there were fewer moults, supporting the idea that oxygen availability directly influences developmental processes.

External resource conditions, such as environmental hypoxia or food conditions, appear to affect development less compared to their effects on growth rates. Development rate is generally more sensitive to temperature than growth rate, and this temperature dependence also appears to vary less across ontogeny (e.g. Horne *et al*., 2019). In addition, stimulating effects of temperature on growth seemed to level off with increasing temperature (De Block & Stoks, 2003) and increasing body size during ontogeny (Forster *et al*., 2012), which suggested resource limitation (or responses to avoid it; see Section IV.2) under these conditions. By contrast, thermal effects on development did not suggest resource limitation or its avoidance. Changes in development rates across populations occupying different positions along a latitudinal or altitudinal cline suggest adaptive modulations of development. Substantial counter‐gradient variation in development rate has been reported across latitudinal and altitudinal clines (e.g. faster development of high‐latitude populations), likely as an adaptation to the shorter growing season at high altitudes and latitudes (Ayres & Scriber, 1994; Dingle & Mousseau, 1994; Chown & Klok, 2003; Blanckenhorn & Demont, 2004; Berner & Blanckenhorn, 2006; Kivelä *et al*., 2011; Parson & Joern, 2014; Buckley *et al*., 2015). Co‐gradient variation in development rate has also been reported, but again to resolve time limitations (i.e. faster development in warmer, but ephemeral habitats) (Dittrich *et al*., 2016). Heliotherms prefer and reach high operative body temperatures *via* basking. This could select for a reduced development rate, or a lower thermal sensitivity of development which avoids leaving insufficient time for completing growth in terms of mass, thus explaining converse TSR in heliotherms (see Section IV.6). In summary, responses of development rate to temperature appear to be adapted to duration of the growing season. Compared to growth, development is relatively insensitive to availability of environmental resources.

### (6) Are grasshoppers an exception to the temperature–size rule?

A notable exception to the near‐universal pattern of size reductions with warming are the grasshoppers. Grasshoppers could be less inclined to follow the TSR for several reasons.

First, oxygen may be less limiting in larger terrestrial arthropods such as grasshoppers, because gas exchange in their tracheal network relies more on convection (Greenlee & Harrison, 2004). This could at least partially explain why they differ from aquatic counterparts, but is unlikely to be the complete reason, as plenty of large tracheated arthropods do follow the TSR.

Second, grasshoppers are heliotherms and have a high preferred body temperature, sometimes as high as 38°C (Miller *et al*., 2009). Heliotherms will likely also experience larger variations in body temperature than other ectotherms. Under widely fluctuating temperatures, the realized thermal performance curve for growth is different from the thermal performance curve under constant temperatures due to Jensen's inequality (Denny, 2017), reaching peak performance at a lower temperature. To compensate, heliotherms likely have a thermal performance curve with a peak shifted to higher temperatures and since most TSR rearing experiments employ constant temperatures, the higher rearing temperatures will strongly stimulate growth as they are unlikely to coincide with limitations for resource supply. In addition to growth being highly responsive to temperature, development may be less responsive to temperature in heliotherms: the operative body temperatures of heliotherms may have frequent excursions into the warmer ranges of their thermal window, and a low thermal sensitivity for development may be required to prevent development from proceeding too rapidly, which would leave little time for the animal to grow. As argued in Section IV.7, a low thermal sensitivity of development rate appears to be associated with larger genomes, and grasshoppers indeed have the largest genome among insects (Alfsnes, Leinaas, & Hessen, 2017). The combination of a high thermal sensitivity for growth rate (at the rearing temperatures employed) and a reduced thermal sensitivity for development rate will make a positive T–S response more likely in grasshoppers and other heliotherms (e.g. lizards).

Grasshoppers may adaptively reverse the TSR for other reasons. First, sun‐basking grasshoppers will gain heat rapidly, but heat loss will be equally rapid as they are too small to conserve heat in any significant amount. According to the heat balance model by Olalla‐Tárraga & Rodríguez (2007), it could be adaptive to be smaller in colder environments: during the periods of sunshine they can then heat up more rapidly and spend less time in absolute terms on heating up and more time on foraging.

Second, grasshoppers are commonly univoltine owing to an obligatory diapause in their egg stage (Van Wingerden, Musters, & Maaskamp, 1991), making it more profitable to grow larger, as completing an additional generation may not be an option. Larger adult grasshoppers produce proportionately larger egg pods, conferring a fitness advantage to growing larger (Walters & Hassall, 2006). In warmer conditions, avoidance of excessive developmental acceleration would leave sufficient time for the animal to grow to a large and fecund body size (Berner & Blanckenhorn, 2006). In line with this reasoning, Buckley *et al*. (2015) documented that grasshoppers inhabiting high elevations increased their development time over the course of 50 years of climate warming.

### (7) Effects of cell and genome size on thermal responses

Changes in body size mostly result from changes in either cell number, cell size, or a combination of these (Calboli, Gilchrist, & Partridge, 2003). As a result, thermal plasticity in body size could reflect changes in cell size (Hessen, Daufresne, & Leinaas, 2013; Table 1). Clearly, changes in cell size mirror changes in body size in eutelic animals, whose number of cells upon reaching adulthood is fixed (e.g. rotifers, most nematodes and some copepods; see McLaren & Marcogliese, 1983; Ruppert, Fox, & Barnes, 2004). However, also in non‐eutelic animals, changes in cell size can correlate strongly with T–S responses (Partridge *et al*., 1994; Van Voorhies, 1996; Arendt, 2007; Hermaniuk, Rybacki, & Taylor, 2016; Leinaas *et al*., 2016). Strikingly, while food availability generally affects cell number, temperature appears to act mainly *via* changing cell size (Arendt, 2007; Czarnołęski *et al*., 2013), although the effects of food and temperature are not completely independent (Padmanabha *et al*., 2011). Thus T–S responses at the cellular level are also consistent with the contrasting effects of rearing temperature and food conditions on whole‐organism size (Berrigan & Charnov, 1994).

Across species or degrees of cell ploidy, cell size appears to be linked to the size of the nucleus, which in turn is linked to genome size, although the causality and its direction are not completely resolved (Gregory, 2001; Cavalier‐Smith, 2005; Hessen *et al*., 2013). Indeed, artificially inducing triploidy in zebrafish (*Danio rerio*) resulted in a 50% increase in cell size, resembling the 50% increase in genome size (Van de Pol, Flik, & Verberk, 2020). Studies have found that plastic thermal responses in body size were accompanied by dynamic adjustments in both cell size and nucleus size (by adjusting chromatin packaging) and thus there is scope for cell size also to generate or parallel the TSR during ontogeny (Hermaniuk *et al*., 2016; Leinaas *et al*., 2016).

The consequences of cell size are temperature dependent (Szarski, 1983). Protein synthesis rates are naturally slowed down in the cold. Boosted expression of key enzymes to maintain adequate protein synthesis rates at the cellular level may be facilitated by a large genome with multiple gene copies due to gene duplication, or by having uncondensed DNA (Xia, 1995; Hessen *et al*., 2013). Another potential advantage of larger cells in the cold is to mitigate developmental noise. Finite numbers of molecules [proteins or messenger RNA (mRNA)] introduce stochasticity in developmental pathways whose regulation arising from random interactions of molecules becomes increasingly unpredictable and variable with reduced absolute numbers of molecules (see Woods, 2014). Such stochasticity increases if the number of molecules that participate in a reaction are lower or if the reaction rates are slower. Thus, having larger cells with higher absolute numbers of molecules mitigates the effect of slower reaction rates in the cold. Differences in cell size could also be mechanistically linked to oxygen supply (Woods, 1999; Makarieva *et al*., 2005; Atkinson *et al*., 2006). Since diffusion rates of oxygen are greater in lipids, membranes may act as preferential diffusion pathways for oxygen (Subczynski, Hyde, & Kusumi, 1989). Small cells also have more surface area relative to volume, conferring a greater capacity for uptake of oxygen and other resources. Finally, diffusion distances from the cell membrane to the mitochondria in the cytosol are smaller in small cells. A reduced cell size in warm conditions may thus be part of an adaptive response to improve oxygen provisioning and modulate growth and development. Reductions in cell size might be achieved *via* oxygen sensing and activation of the HIF (hypoxia‐inducible factor) and mTOR (mammalian target of rapamycin) regulatory pathways (e.g. Guzy & Schumacker, 2006). In the nematode *Caenorhabditis elegans*, a mutation in a single gene appeared to control whether animals conformed to the TSR or not (Kammenga *et al*., 2007). The gene involved encoded a calpain‐like protease, which has a high homology with mammalian calpains known to regulate cell size and which can be induced by hypoxia (Cui *et al*., 2015). An oxygen perspective may therefore apply not just to organism size, but also to a lower, cellular, level of biological organization, whereby a reduced capacity for oxygen uptake may impact the energy budgets of larger cells (Atkinson *et al*., 2006; Table 1).

The strength and direction of T–S responses could also be related to cell size. If (temperature‐induced) risks of oxygen limitation are more likely to arise in tissues made up of large cells, animals with larger cells may be more likely to reduce cell size plastically to improve oxygen provisioning. If variation in cell number is small, such changes in cell size will be reflected in stronger T–S responses in body size. Triploid tadpoles of the frog *Pelophylax esculentus* were indeed shown to exhibit a stronger T–S response upon metamorphosis compared to diploid tadpoles (Hermaniuk *et al*., 2016) and also a comparison of fruit flies differing in genome size revealed stronger T–S responses in flies with larger genomes (Ellis *et al*., 2014). Wyngaard *et al*. (2005) reported differences in T–S responses across five species of copepods whereby the strongest T–S responses were observed in the species with the largest genome [*M. latipes*: ~ C‐value (the amount, in picograms, of DNA contained within a haploid nucleus) of 4 pg or 3.91 × 10^9^ base pairs], and the weakest T–S responses were observed in the species with the smallest genome (*T. crassus*: ~ 0.8 pg). Horne *et al*. (2016) report differences in T–S responses in which copepods of the order Calanoida (~4.5 pg) showed a stronger TSR than those of the order Cyclopoida (~ 1 pg). These orders exhibit a significant difference in genome size (and hence probably cell size) [*t*‐test: *P* = 1.005 × 10^−7^; calculated from data in Gregory, 2018; see also Wyngaard & Rasch, 2000]. Similarly, aquatic species living in cold environments tend to have larger genomes (Dufresne & Jeffery, 2011; Lorch *et al*., 2016; Alfsnes *et al*., 2017; Jeffery, Yampolsky, & Gregory, 2017), suggesting a cell‐size parallel with Bergmann's rule, at least for aquatic animals. In general, animals increase body size mainly through cell proliferation during early development, but by cell growth in later life (Kammenga *et al*., 2007; Czarnołęski *et al*., 2008, but see Aguilar‐Alberola & Mesquita‐Joanes, 2014; Horne *et al*., 2019) and this fits with the TSR being less pronounced for egg size, manifesting itself in later life stages (Forster, Hirst, & Atkinson, 2011; Forster & Hirst, 2012). In summary, there are clear patterns between genome size and the strength of the T–S response, with stronger T–S responses being found in animals with larger cell sizes. Such patterns suggest a link between cell size and the strength of the TSR.

Genome size is also linked to development rate, with large genomes being associated with slower development in fruit flies (e.g. Gregory & Johnston, 2008), copepods (McLaren, Sevigny, & Corkett, 1988) and anurans (van der Have, 2008). Genome size and development rate are mechanistically linked as DNA replication takes proportionally longer with larger genomes (Van't Hof & Sparrow, 1963), although slow replication of large genomes can be compensated for by increased ribosomal DNA (rDNA) copy number (White & McLaren, 2000; Prokopowich, Gregory, & Crease, 2003). These genomic effects on development rates may be temperature specific (e.g. Ellis *et al*., 2014), and a lower thermal sensitivity of development has been reported in copepod species with a larger genome (Wyngaard *et al*., 2005), and in both triploid froglets (Hermaniuk *et al*., 2016) and polyploid cladocerans (Dufresne & Hebert, 1998; Van Geest *et al*., 2010) when compared to their diploid counterparts. Whereas a lower thermal sensitivity of development may be beneficial in certain environments (see Section IV.6), several copepods exhibit chromatin diminution during early embryogenesis, possibly as a way to increase development rate by removing the burden of lengthy replication cycles from large genomes. Chromatin diminution results in substantial decreases in nuclear DNA content of the somatic cells (Wyngaard & Rasch, 2000) due to chromosomal fragmentation and excision of large portions of DNA in the presomatic line. Such diminution has also been reported in other eukaryotes (Parfrey, Lahr, & Katz, 2008). In summary, there is evidence that a large genome size reduces development rates and possibly also their thermal sensitivity.

Walczyńska *et al*. (2015a, 2015b), building on the work of Stelzer (2002), showed that in eutelic rotifers, thermal responses in cell size (and thus body size) followed the TSR. In addition to the absolute temperature, the direction in which temperature changed mattered. When reared at a common temperature of 20°C, differences in cell size depended on the temperatures the mothers had previously experienced during egg development: offspring from mothers that had experienced warmer conditions attained a larger cell size, compared to offspring from mothers that had experienced cooler conditions. Egg size in fruit flies (Crill, Huey, & Gilchrist, 1996) and butterflies (Fisher *et al*., 2003) was also found to vary in response to the temperature that parents experienced. The importance of parental temperature and the direction of temperature change suggest that parents convey information to their offspring on when to arrest cell growth. Thus, it is unlikely that oxygen or another resource sets absolute or insurmountable limits to the size that a cell can attain (see also Section IV.2); cells will not keep growing to the point at which they will become energy limited due to insufficient resource provisioning. Since conditions experienced by the parents can reasonably be anticipated to resemble the conditions that the offspring will face as well, providing such information may be adaptive. If offspring developed at temperatures cooler than those experienced by the parents, offspring had larger cells and *vice versa* when temperatures are warmer than parental temperatures (Walczyńska *et al*., 2015*b*). Documented responses in body size to temperature and oxygen combinations coincided with higher fecundity, suggesting that they are adaptive (Walczyńska *et al*., 2015*a*). Thus, the information bestowed upon the offspring may arrest cell growth when the ratio between oxygen supply and demand falls below a certain threshold. The ratio between oxygen supply and demand is reduced both by an increase in cell size (by reducing oxygen supply) and an increase in temperature (by increasing oxygen demand). Such a threshold ratio may safeguard sufficient oxygen provisioning under warmer conditions (Walczyńska *et al*., 2015a, 2015b). A similar threshold may govern the critical size in insect development; critical size decreases both under warming (Ghosh, Testa, & Shingleton, 2013) and hypoxia (Callier *et al*., 2013), while there is also evidence for increased critical size under hyperoxia (Kivelä *et al*., 2018). Also, when medaka fish (*Oryzias latipes*) were reared for multiple generations, the smallest size was observed in fish reared under temperatures that were warmer than they had previously experienced (Loisel *et al*., 2019). A tentative conclusion from the studies reviewed above is that oxygen limitation may take the form of an ultimate driver, whereby animals have evolved plastic, canalized responses geared to avoid oxygen limitation, limiting cell growth to a point with sufficient capacity for oxygen provisioning, a threshold calibrated against the temperatures experienced by adults [see also Harrison *et al*., 2018 and Section VI.3].

### (8) Explaining the TSR as the balance between growth and development

**Fig 4:**
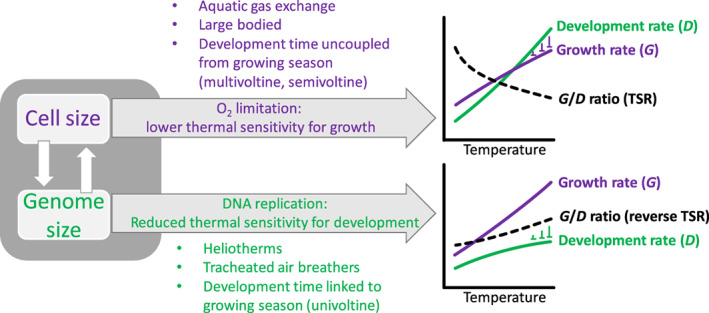
Overview of influences on growth rate (*G*) and development rate (*D*) responses to temperature, and hence their ratio and the temperature–size response. TSR, temperature–size rule. Temperature stimulates both growth rate and development rate, but the relative increase may be modulated by effects of cell size, genome size, body size, life cycle, thermoregulatory behaviour and mode of respiration. Oxygen limitation is more likely in large aquatic ectotherms with large cells, and could constrain the stimulating effects of temperature on growth rate. Consequently, animal development outpaces growth under warmer conditions, resulting in a decrease in body size (purple pathway). A large genome size may be associated with a lower thermal sensitivity of development. Consequently, development does not outpace growth under warmer conditions and the faster growth results in larger body sizes (green pathway). Due to the strong linkage between genome size and cell size, both mechanisms will operate in tandem, but the relative importance of these mechanisms may differ among animals, depending on their characteristics.

A low thermal sensitivity of development rate relative to growth rate weakens (or reverses) the TSR, while the TSR is strengthened if development rates increase with temperature more than do growth rates. We have seen why warming‐induced acceleration is more likely curtailed for growth: although limited oxygen or food is unlikely to constrain growth rates directly when provided *ad libitum*, growth rates could be adaptively modulated as a result of warming exacerbating resource limitation during the species’ evolutionary history (see Section IV.2). Risks of oxygen limitation are more likely in ectotherms that rely on underwater gas exchange, but are less likely in air‐breathing, tracheated arthropods that employ convective ventilation. Since the TSR is expressed at the level of the whole organism (size at maturity, asymptotic size), it integrates the effects that strengthen or weaken the TSR at each level of biological organization. A large genome can either weaken the TSR if it predominantly decelerates developmental rate, or strengthen the TSR when the concomitant larger cell size results in oxygen limitation effects on growth (Fig. 4). With oxygen limitation likely having less of an influence in air‐breathers, the effect of genome size on development rate could be dominant here. The slowing down of development rates with increases in genome size could explain why latitudinal clines of genome size are predominantly negative for (terrestrial) insects (see Alfsnes *et al*., 2017): at higher latitudes the shorter seasons would require smaller genomes to enable more rapid development. By contrast, for aquatic arthropods cell‐size effects on oxygen limitation may be more important under warm conditions. This could help explain the divergent T–S response between these two groups.

## V. WHY ARE NEGATIVE T–S RESPONSES SO PREVALENT?

Assuming that the TSR is adaptive, resources are allocated in a temperature‐ and size‐dependent way to enhance fitness. Compared to cool conditions, warmer conditions are associated with faster growth, increased mortality and faster maturity at a smaller size, mirroring major patterns in life histories of animals (Pianka, 1970). The trade‐off between adult size and development time (Abrams *et al*., 1996) places animals on a continuum from early maturation at a small size (allocating resources preferentially to development, prioritizing time), to maturing later at a large size (allocating resources preferentially to growth, prioritizing size). Although there are clear benefits to both being large and being fast, we here focus on how warm temperatures may tip the balance in favour of growing faster to a smaller size and *vice versa* (Table 1).

### (1) Mortality and reproduction are temperature‐ and size‐dependent

Prioritizing time may be favoured in warm conditions, as warming may reduce lifespan *via* increased competition, predation or resource scarcity (food or oxygen). Such increased mortality risks may be associated with thermal acceleration of physiological rates (e.g. growth, development and reproduction), whose resultant energetic costs are known to impair immune function (De Block & Stoks, 2008) and reduce lifespan (Lee, Monaghan, & Metcalfe, 2013; Lind *et al*., 2017), likely *via* oxidative stress and cellular senescence (Hemmer‐Brepson *et al*., 2014). Increased mortality typically favours adaptively reducing the duration of the life stage at increased risk. In addition, if warm conditions during juvenile growth incur costs that increase the risk of (reproductive) senescence or reduced lifespan, it should pay to reproduce sooner (with consequently smaller size) to reduce these risks, which thus can provide an adaptive explanation for the TSR at maturity (Sibly & Atkinson, 1994; Kindlmann *et al*., 2001; Kozłowski *et al*., 2004). Thermal effects on survivorship reported from laboratory studies do not generally constitute a sufficiently strong selection pressure to account fully for the TSR (Myers & Runge, 1983; Angilletta, Steury, & Sears, 2004). However, the TSR may still be explained adaptively from increased mortality at higher temperatures if thermal effects in the field are larger than those reported from laboratory studies, for example when higher temperature increases predator‐induced mortality (Hirst & Kiorboe, 2002). Increased mortality risks in the warm may also be size dependent (e.g. related to predator escape). If mortality increases with warming especially in larger individuals as has been found for *Daphnia magna* (Bruijning, ten Berge, & Jongejans, 2018), it could be beneficial to mature at a smaller size. Leiva, Calosi, & Verberk (2019) also found survival of heat stress to be dependent on body size.

Prioritizing size may be favoured in cold conditions because of gains in fecundity. Larger mothers typically produce more offspring. In fish, fecundity, egg size and egg energy content all increased with body size, such that larger mothers had disproportionately higher reproductive energy output (Barneche *et al*., 2018). Such gains in fecundity in larger individuals were magnified under cold conditions in freshwater snails of the genus *Physa* (Arendt, 2015) and in *Daphnia* cladocerans (Weetman & Atkinson, 2004), but not in the water strider *Aquarius remiges* (Arendt & Fairbairn, 2012). In summary, temperature may evolve as a cue such that warm conditions accelerate juvenile development rate, because of a predictable association between warm conditions and increased mortality risks in the field during a species’ evolutionary past or because the size–fecundity relationship changes with temperature.

### (2) Resource limitation is temperature‐ and size‐dependent

At higher temperatures, ectotherms require more resources to fuel their enhanced activity rates. Although this increased demand may not constrain growth (see Section IV.2) it could increase the risk that resources (e.g. oxygen or food) become limiting under resource‐demanding conditions. Therefore, negative T–S responses could have evolved to avoid resource limitation. This hypothesis can be considered the selective effect of ‘resource‐limitation past’ (see Section VI.3 for this principle applied to oxygen). Larger individuals have a higher *per capita* resource demand and both their aerobic scope and their ability to obtain sufficient food or oxygen may be less capable of matching warming‐enhanced demand compared with that of smaller individuals (Atkinson *et al*., 2006; Neubauer & Andersen, 2019; J.G. Rubalcaba, W.C.E.P. Verberk, A.J. Hendriks, B. Saris. & H.A. Woods, in preparation). Although larger individuals can have greater tolerance to lack of food (Cushman, Lawton, & Manly, 1993; Arnett & Gotelli, 2003; Scharf, Galkin, & Halle, 2015), starvation is more likely outside the growing season (i.e. winter). Moreover, studies demonstrating that animals evolve larger body sizes when reared under cool conditions and fed *ad libitum* (e.g. Partridge *et al*., 1994) indicate that starvation is not a necessary condition for the evolution of the observed T–S reaction norms. In summary, temperature may evolve as a cue such that cool conditions favour growing to a larger size, because of a predictable association from the species’ evolutionary past, between cool conditions and lower risks of resources becoming limiting.

### (3) Mortality and fecundity are often resource dependent

Evolutionary effects of mortality, fecundity (see Section V.1), and resource limitation (see Section V.2) are not independent: resource shortage can impair fecundity and increase mortality risks. Hence, seemingly disparate causes of the TSR can be unified. Specifically, although some warming‐enhanced mortality risks (e.g. predation, pond drying) may kill irrespective of resource availability to the organism, it is clear that mortality and resource limitation (related to an organism's aerobic scope, feeding capacity or energy reserves) are often interdependent. On the one hand, increased resource limitation may limit the ability to mount a sufficient defence against threats such as predators or drought, and hence lead to increased mortality from these threats. This increased mortality could favour the evolution of a reaction norm that accelerates juvenile development (maturation) in the warm, thereby reducing this resource‐dependent mortality (see Section V.1). On the other hand, selection to actively avoid increased predation risk or unfavourable environmental conditions in the warm may increase selection for a greater resource‐supply safety margin, thereby favouring the evolution of a thermal reaction norm producing individuals with a larger safety margin (e.g. aerobic scope), which may be achieved by growing to a smaller size (see Section IV.2). In both cases, adults would be smaller, and would mature earlier in the warm. Thermal effects on fecundity are also likely to be highly dependent on the resource‐supply safety margin for adults of different sizes. For instance, in the (aquatic) rotifer *Lecane inermis*, gains in fecundity with body size were dependent not only on temperature, but also on oxygen levels (Walczyńska *et al*., 2015*a*), and in mosquitofish (*Gambusia affinis*) populations, the reproductive advantage of larger body size decreased with increasing site temperature within 100 years (Fryxell *et al*., 2020).

In summary, the hypotheses discussed in Sections V.1 and V.2 are not independent. Indeed, to explain the TSR, it is not necessary for resource limitation, mortality and fecundity all to be size and temperature dependent. If one of these is, and their effects are amplified by any of the others, a TSR response can evolve from a variety of mechanisms, potentially explaining why it is so widespread among ectotherms.

## VI. WHAT EXPLAINS MOST OF THE VARIATION IN T–S RESPONSES?

Although adult body size is usually reduced under warmer rearing conditions, understanding the causes of variation in the magnitude and direction of T–S responses is likely to lead to a more complete explanation of why body size changes with temperature. The most striking differences to be explained occur between aquatic and terrestrial species (Forster *et al*., 2012; Horne *et al*., 2015). Here we focus on the greater thermal seasonality in terrestrial environments, stronger selection to avoid oxygen limitation in aquatic environments, and temperature being a more reliable cue in aquatic environments.

### (1) Seasonality and voltinism

**Fig 5:**
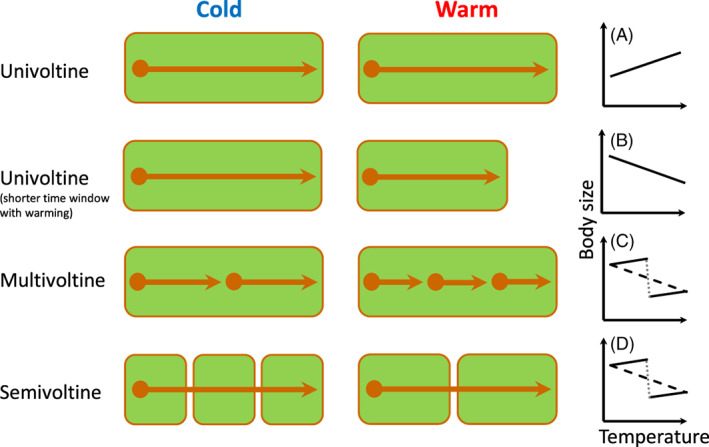
Schematic overview of different temperature‐size (T–S) responses in relation to voltinism. T–S responses may depend on the interaction between the length of the growing season (green box) and the development time (brown arrow), especially in (terrestrial) organisms living in habitats with strong end‐of season constraints. For univoltine species, warming may allow animals to grow faster during their (fixed) development time, resulting in animals reaching a larger size (A), unless time for development is also reduced under warmer conditions (B). Warming may also allow animals to fit more generations into a certain amount of time, either by increasing the number of generations (C; multivoltine species) or by decreasing the number of years needed for completion (D; semivoltine species). A faster development can result in animals growing to a smaller size under warmer conditions when viewed across the whole thermal gradient (dashed black line). However, shifts in voltinism may result in a sawtooth pattern, with animals growing to a larger size with warming (solid black line), until there is an increase in voltinism at which point animals reach a smaller size (due to less time available for growth in a given generation, dotted grey line). Such shifts in voltinism and the resulting sawtooth patterns are most readily seen in latitudinal clines.

Maturing earlier may become feasible under warmer conditions (e.g. due to enhanced growth and food consumption). The benefits of early breeding or even completion of an extra generation may be substantial (Cole, 1954), and can outweigh the disadvantages associated with a smaller body size. These benefits of early maturation are greater in growing populations, since the earlier an organism breeds the greater the proportional contribution each offspring makes to the population. This increase in parental fitness is analogous to the increased financial gains from investing money early in a bank account that gives high compound interest (Calow, 1981; Kozłowski, 1992; Atkinson, 1994; Fischer & Fiedler, 2002; Kingsolver & Huey, 2008). Although population growth rate is variable and not consistently linked to temperature, in seasonal habitats, the number of generations in a year may influence T–S responses. Univoltine species, completing a single generation every year, do not have the opportunity to increase fitness by speeding up development to increase the number of generations per year, especially when univoltism is enforced by an obligatory diapause. Instead, obligate univoltine species should use all the time available to them, and their faster growth in warm conditions therefore will weaken the TSR or even give rise to a converse TSR (Fig. 5; overcompensation *sensu* Blanckenhorn & Demont, 2004). Indeed, Fischer & Fiedler (2002) demonstrated a weaker TSR in univoltine populations of the butterfly *Lycaena hippothoe* than in multivoltine populations. Similarly, perceived time available can alter T–S responses (see Section VI.2). Moreover, Sniegula, Golab, & Johansson (2016) reported a smaller size at maturity in cold‐latitude populations of the obligate univoltine damselfly *Lestes sponsa* (i.e. a converse James’ cline). Grasshoppers have a diapause at the egg stage, enforcing univoltism, which may explain why grasshoppers generally exhibit converse T–S responses (see Section IV.6). Some univoltine species may be obligatorily univoltine not because of a limited duration of the warm season *per se*, but because host plants are available for a specific period only, such as is the case for the butterfly *Anthocharis cardamines* (Posledovich *et al*., 2014). When host plants are available for a shorter period due to warming, this may still result in faster development to a smaller size despite being univoltine (Fig. 5). Semivoltine species, like multivoltine species, can decrease the time spent per generation (in their case by decreasing the number of years per generation): benefits from increasing numbers of generations per unit time can therefore accrue to species from both groups, but not to univoltine species (Fig. 5). In summary, options to accrue fitness by speeding up development are limited in univoltine species and these are therefore less likely to adhere to the TSR.

Seasonality interacts with voltinism as strong end‐of season constraints will result in high mortality, selecting against semivoltine species taking multiple years to complete a generation (Walczyńska, Dańko, & Kozłowski, 2010; Ejsmond *et al*., 2018). Chown & Klok (2003) found clear differences in altitudinal clines in body size between two regions that differed substantially in seasonality. Species in the region with strong end‐of season constraints displayed discrete generations and converse size clines (larger individuals at lower, warmer altitudes), whereas those in the region without clear seasonality displayed overlapping generations and achieved the largest body size in colder, higher altitudes. Minards *et al*. (2014) also report differences between low‐ and high‐altitude populations of *Hemideina* orthopterans in their T–S responses established by rearing animals in the laboratory: low‐altitude populations followed the TSR and high‐altitude populations showed a converse T–S response, suggesting that thermal responses may be adaptively shaped by selection due to differences in season length. The importance of end‐of‐season constraints and voltinism is also suggested in a study of community‐level body‐size gradients by Zeuss *et al*. (2017): aquatic odonates showed Bergmann clines, whereas terrestrial lepidopterans showed converse Bergmann clines. The different responses of lepidopterans and odonates could at least partly be explained *via* effects on voltinism: many odonate species extend juvenile development over multiple years, whereas most butterflies were univoltine and hence more likely to have seasonal time limitation on body size, which would be relaxed at lower latitudes with longer annual growth periods. In summary, the risks of not completing juvenile development in time before the onset of winter has much more severe consequences in terrestrial environments than in aquatic environments (e.g. Van Dyck *et al*., 2015; Forrest, Regan Cross, & Cara Donna, 2019): this may explain why time constraints did not generally reverse T–S responses of aquatic ectotherms (Cabanita & Atkinson, 2006), and may contribute to the difference between aquatic and terrestrial T–S responses (Forster *et al*., 2012; Horne *et al*., 2015; Rollinson & Rowe, 2018).

### (2) Effects of day length on T–S responses

To understand seasonality effects in the field better, laboratory experiments have shown how day length modulates the effects of temperature on size, changing the allocation of resources to growth, development, reproduction and maintenance (lifespan) (Ernsting & Isaaks, 2000; Camus & Zeng, 2008). Consequently, day length may affect the strength of the TSR in both terrestrial and aquatic taxa (e.g. Kutcherov *et al*., 2011; Martínez‐Jerónimo, 2012; Kollberg *et al*., 2013; De Block & Stoks, 2003; but see Cabanita & Atkinson, 2006). In multivoltine tephritid flies, warming results in early maturation at a smaller size under an early‐season photoperiod, whereas maturation was delayed under a late‐season photoperiod, resulting in larger adults with presumably better chances of surviving winter conditions (Xi *et al*., 2016). Such modulation of T–S responses and of juvenile development period by photoperiod suggests that these animals use day length as a cue to gauge time in the year, and hence the availability of future favourable conditions. Animals tend to speed up development when less time is available, but they can also increase growth rates, which can buffer changes in adult size (Abrams *et al*., 1996; Blanckenhorn & Demont, 2004; Kivelä *et al*., 2011; Buckley *et al*., 2015). This complexity between voltinism, body size and temperature can make it difficult to disentangle the different influences (Cabanita & Atkinson, 2006) and males and females may prioritize development time and body size differently (De Block & Stoks, 2003). What is clear though is that cold, high‐latitude environments present stronger time limitations, which may be overcome by extending development over multiple years (decreasing voltinism). Changes in voltinism across populations may be accompanied by changes in allele frequency of clock genes linked to post‐diapause development time such that complete life cycles can be ‘fitted’ into latitudinally varying growing seasons (Levy *et al*., 2015). Shifts in voltinism across thermal clines can result in changes in strength of seasonal time constraints on the populations. Consequently, thermal responses in body size along thermal clines (e.g. associated with altitude and latitude) can be discontinuous, following a saw‐tooth pattern, which arises from alternate intensification and relaxation of time constraints as both voltinism and season length vary (Roff, 1980). Within species of aquatic arthropods, gradients of body size across latitudes are non‐linear, indicating that there is more to latitudinal clines in body size than just temperature (Johansson, 2003; Hassall, 2013). In summary, day length may alter the thermal reaction norms and the resulting size and age at maturity in ways that depend on: (*i*) the voltinism of the population from which the individuals originated; (*ii*) the physiological state of the individual; and (*iii*) the temperature and light regime of its environment (Honĕk, 1996; Gotthard, Nylin, & Wiklund, 2000; Lopatina *et al*., 2011; Martínez‐Jerónimo, 2012; Clemmensen & Hahn, 2015). All of these provide information about availability of time for development (Roff, 1980; Lee, Monaghan, & Metcalfe, 2010).

### (3) The ‘Ghost of Oxygen‐limitation Past’

The proximate mechanisms by which a shortage of oxygen puts large individuals in the warm at a disadvantage, especially in aquatic environments (see Section IV.3) can also act on evolutionary timescales to eliminate phenotypes that produce large adults under warm conditions. Importantly, powerful selective events in the past need not be frequent to affect the phenotypes of descendants (Grant *et al*., 2017). According to this evolutionary perspective, at warmer temperatures oxygen may be limiting only infrequently, even for aquatic species, because past selection on T–S responses has eliminated genotypes more prone to oxygen limitation. This selection could favour genotypes with enhanced supply capacity, and indeed, arthropods have evolved a suite of plastic responses geared to avoid oxygen limitation (see Harrison *et al*., 2018). For aquatic ectotherms, selection may also have favoured reaction norms that increase body size in cool conditions, as being large may be helpful in overcoming the viscosity of cold water: larger animals can generate higher flow speeds of water, which increases the energy efficiency of gill surface irrigation and of body propulsion (Verberk & Atkinson, 2013). Experiments that investigate T–S responses typically include normoxic laboratory settings with abundant food and without natural enemies. Under these favourable conditions, individuals would be unlikely to experience resource limitation. Instead, these individuals would have evolved a canalized growth response that safeguards sufficient oxygen provisioning (e.g. a safety margin, such as aerobic scope) under warmer conditions. In the same way that Connell's ‘Ghost of Competition Past’ could explain how avoidance of competition had evolved (Connell, 1980), we can invoke a ‘Ghost of Oxygen‐limitation Past’ potentially to explain the evolution of a strong TSR in aquatic species, even if oxygen limitation is not evident under favourable conditions. This idea can explain why responses to hyperoxia are usually small (Frazier *et al*., 2001): the ‘Ghost of Oxygen‐limitation Past’ may have selected against animals that would grow bigger in the presence of additional oxygen if their ancestors did not benefit from an increased body size at hyperoxia. Indeed, fruit fly body‐size responses to varying levels of oxygen do evolve (Henry & Harrison, 2004). T–S responses also evolve as shown by multi‐generation experiments on medaka fish reared under different temperatures (Loisel *et al*., 2019). Fish reared under warm conditions grew to a smaller asymptotic size compared with those reared under cool conditions. However, fish reared under warm conditions for a single generation grew smaller but also produced fewer offspring than those reared for multiple generations under these conditions (Loisel *et al*., 2019). Thus, evolution can modulate growth trajectories. If the juvenile growth temperatures carry information on whether resource limitation is likely to constrain fitness once these juveniles mature, thermal reaction norms for size at maturity could reflect the balance of oxygen demand to supply under T–S combinations experienced by ancestors (Atkinson & Sibly, 1997*a*; Atkinson *et al*., 2006). In this way, temperatures experienced early in ontogeny may act as a cue to adjust growth trajectories and the resulting adult or final size. Such cues may also be experienced by the mother, and this information can then be passed on to the offspring as has been argued for adaptive changes in cell size [see Walczyńska *et al*., 2015b and Section IV.7].

### (4) Temperature and oxygen as an information cue

The ‘Ghost of Oxygen‐limitation Past’ views T–S responses as adaptive responses to maintain a safety margin for aerobic scope (Fig. 3) that have evolved in response to temperature and oxygen conditions experienced by ancestors. These reaction norms incorporate temperature and oxygen levels (or physiological correlates of oxygen levels) as cues and the information content of such cues is likely to differ between terrestrial and aquatic environments. In aquatic environments, temperature fluctuates less and varies more predictably than on land, especially for larger bodies of water, making water temperature a reliable cue. Moreover, terrestrial animals can exploit the greater thermal heterogeneity on land *via* behavioural thermoregulation, reducing the information content of air temperature. Hypoxia is also more common in aquatic environments, especially during warm periods. The ‘Ghost of Oxygen‐limitation Past’ is therefore more likely to operate in aquatic ectotherms, favouring genotypes that grow to a smaller size in warm water, and consequently avoiding oxygen constraints on growth. This prediction is consistent with the stronger TSR in aquatic than in terrestrial species (Fig. 2; Forster *et al*., 2012; Horne *et al*., 2015). In summary, on land, temperature is spatially more heterogeneous and temporally more variable. Coupled to the ability to thermoregulate, this may weaken any selection pressure on thermal reaction norms for size and age at maturity in terrestrial species.

## VII. QUESTIONS AND DIRECTIONS FOR FUTURE RESEARCH

We are beginning to understand the distinct patterns in T–S responses across animal groups. Still, many important questions remain, and here we propose some questions and directions for future research:

1. Elucidate the role of cell size and genome size in generating the TSR. Does the thermal dependency of growth and development differ with cell size or genome size in both terrestrial and aquatic ectotherms? How do cell proliferation and cellular enlargement contribute to whole‐organism growth during ontogeny, and do T–S responses become stronger during periods where cellular enlargement contributes most? Is the decision to arrest cell growth governed by a threshold ratio between supply and demand of oxygen to safeguard sufficient oxygen delivery, which is calibrated against the temperatures experienced by parents? How does this threshold ratio relate to the critical size of an insect?
2. Elucidate the role of ecological factors in shaping T–S responses. How do T–S responses change with photoperiod, voltinism, predator characteristics (endotherm, ectotherm, sit‐and‐wait, active hunter, etc.) and food conditions? How do T–S responses affect community assembly by affecting geographic range shifts, predator–prey relationships and phenology?
3. Extend the scope of data on T–S responses. How does developmental temperature affect body size in larger‐bodied species, both aquatic and terrestrial? How does size in aquatic species change across altitudinal clines? How do T–S responses change over multiple generations when long‐term adaptation also starts to play a role? Do multiple‐generation T–S responses differ if temperature is decoupled from proposed selective factors (e.g. oxygen supply, time constraints, mortality schedules)?
4. Create a database for T–S responses, facilitating future (meta) analyses.
5. Explain the adaptive nature of T–S responses by integrating growth trajectories of juveniles to fitness consequences of adults (e.g. by modelling of energy budgets or conducting multigenerational studies into the evolution of T–S reaction norms, where putative selective agents (e.g. oxygen limitation, mortality risks) are decoupled from temperature. Are larger individuals more susceptible to oxygen limitation under warmer conditions and does such (incipient) oxygen limitation proximately or ultimately limit growth?

## VIII. CONCLUSIONS

1. The many explanations proposed (Table 1) for observed phenotypically plastic body‐size responses to temperature (the TSR) differ in domain (focussing on physiological mechanisms that bring about T–S responses or their adaptive nature) and apply to different biological levels of organization (activity rates of enzymes, cells, organisms, populations, communities). The TSR is not universal, but the strength of the TSR varies in predictable ways (being stronger in larger, aquatic ectotherms and being weaker or reversed in larger, terrestrial ectotherms).
2. Effects of temperature are pervasive, affecting biological levels of organization ranging from whole‐organism growth performance down to activities of individual proteins, which makes it unlikely that a single proximate mechanism underlies the TSR. An oxygen perspective may help to explain the effects of temperature on size, especially in large aquatic ectotherms, which are arguably most susceptible to risks of oxygen limitation.
3. Warming may exacerbate risks of oxygen limitation or reduce the safety margin of aerobic scope. For air breathers such as terrestrial insects, problems with insufficient oxygen may be less likely and time constraints take centre stage. Season length may constrain developmental period, forcing them to prioritize time over size as overwintering is more challenging. Day length and temperature may together provide information on how long conditions will remain favourable for development, explaining why thermal responses are modulated by photoperiod.
4. Time constraints, mortality risks, and resource limitation are not mutually exclusive explanations for the TSR. Rather, they may operate in tandem but their relative importance may vary depending on the ecology and physiology of the species in question (Fig. 4). At the level of cells, effects of cell size on oxygen provisioning may be more relevant for aquatic species, whereas effects of genome size on development time may be more relevant for terrestrial species. Similarly, at the level of the whole organism, capacity for oxygen provisioning differs with mode of respiration and habitat use, while end‐of‐season constraints likely differ between aquatic and terrestrial species. Thus, multiple pathways operating at different levels of organization show T–S responses that broadly differ across the aquatic–terrestrial divide. T–S responses may be viewed as being canalized – producing the same adaptive response by a range of mechanisms, since the resultant response has proven its adaptive worth both for safeguarding energy status (e.g. *via* oxygen provisioning) and for safeguarding completion of development (*via* time sensing). As such, oxygen supply can be both a proximate mechanism and an ultimate driver (the ‘Ghost of Oxygen‐limitation Past’).
